# Fern Gametophytes Exhibit Distinct Patterns of Niche Expansion and Convergence in Ecophysiological Functioning

**DOI:** 10.1002/ece3.73324

**Published:** 2026-04-07

**Authors:** Christopher P. Krieg, Jacob L. Watts, Sally M. Chambers, Ameya Baxi, Katherine A. McCulloh

**Affiliations:** ^1^ Department of Biology Wake Forest University Winston‐Salem North Carolina USA; ^2^ Ecology and Evolutionary Biology Department University of Colorado Boulder Colorado USA; ^3^ Department of Biology Eastern Kentucky University Richmond Kentucky USA; ^4^ Department of Biology University of Wisconsin Madison Wisconsin USA

**Keywords:** climate, distribution, ecology, ecophysiology, ferns, gametophytes, niche

## Abstract

Ferns exhibit extraordinary ecological diversity, and their independent gametophyte and sporophyte stages make them distinct among other plant groups, yet the extent to which gametophyte and sporophyte ecology are coordinated is unknown. Here, we integrated spatial and climatic data to test the hypothesis that independent fern gametophytes occupy novel climatic niche space relative to their sporophytes. We also examined the physiological mechanisms in the gametophyte life stage that may underpin spatial patterns of fern ecology. We discovered that 57% of species examined have gametophytes that occupy novel climate niche space relative to their sporophytes and that this expansion occurs along specific axes of climate variation. We also discovered evidence of strong convergence in gametophyte ecophysiological function across broad environmental gradients. In particular, we found that gametophyte photosynthetic rate per mass was strongly linearly correlated to atmospheric water demand (e.g., potential evapotranspiration) and monotonically correlated to precipitation inputs. Taken together, these discoveries represent previously uncharacterized ecology and potential mechanisms that may drive spatial patterns of fern biodiversity.

## Graphical abstract

Some fern gametophytes can explore new habitats that differ from their sporophyte counterparts, and when they do this, they tend to follow specific axes of climate related to aridity (increased or decreased). Moreover, we found that this ecological niche expansion may be driven by ecophysiological adaptations to abiotic environments.

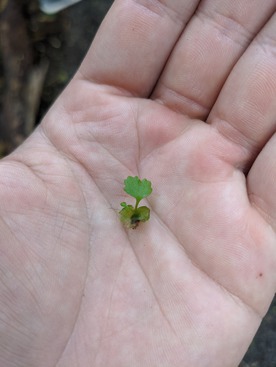

## 1 Introduction

Ecological niche theory has long posited that physiological tolerances and reproductive biology are two primary drivers of spatial biodiversity patterns (Hutchinson 1957; Holt 2009; Ackerly and Monson 2016). Thus, for a species with a given set of physiological traits and reproductive modes, there exists a finite geographic and climatic niche in which the species can survive and successfully reproduce (Hutchinson 1957; Townsend Peterson et al. 2011). Furthermore, competition with other organisms for limited resources may further reduce the available niche space and size of a species' geographic distribution to areas where they can successfully co‐occur with other organisms (Silvertown 2004; Kraft et al. 2015; Karimi et al. 2024). In general, plant species with greater intraspecific physiological and growth variation can exploit a broader set of ecological and climatic conditions, which often lead to a larger geographic range (Darwin 1871; Violle and Jiang 2009; Sides et al. 2014; Carscadden et al. 2020). In addition, many studies have found associations between plant reproductive strategies and habitat preferences, including mosses (During 1979; Grime et al. 1990; Longton 1990), seed‐bearing plants (Matallana et al. 2005; Hargreaves and Eckert 2014; Grossenbacher et al. 2015), and ferns (Peck et al. 1990; Greer and McCarthy 1999; Grusz 2016). Understanding how the coordination of physiological tolerances and reproductive strategies impacts plant distributions is especially critical to predicting how plant biodiversity patterns may change in the future.

The mechanisms by which plants exhibit physiological and reproductive traits that impact their distributions is influenced by evolutionary history. Over evolutionary time, plants have experimented widely with shifts in gametophyte and sporophyte independence. Extant species exhibit a broad macroevolutionary trend in the relative dominance of the gametophyte and sporophyte generation, moving from life cycles with free‐living gametophytes and dependent sporophytes (i.e., bryophytes) to life cycles with dependent gametophytes and free‐living sporophytes (i.e., seed‐bearing plants). The structural and nutritional dependence of gametophyte and sporophytic life stages in these lineages likely exerts a strong influence on modifications to reproduction and/or physiology across these stages (Lenoir et al. 2009; Barrett and Harder 2017; Kupers et al. 2019). Ferns and lycophytes are unique among plant groups because their sporophytes and gametophytes are both free‐living (i.e., independent). Fern sporophytes generally share several anatomical features that impact function and stress tolerance, including laminar cuticles, highly specialized vascular networks, and stomata. This suite of morphological characters keeps them in a state of disequilibrium with their surrounding environment, which allows for effective homeostatic control (Nobel 1978; Pittermann et al. 2015; Sperry et al. 2017; Krieg et al. 2023). In contrast, fern gametophytes are small (generally 1 cm in diameter or less), and survive without a cuticle, vascular tissue, and stomata. Without anatomical features to help individuals dynamically respond to fluctuations in their environment, fern gametophytes are poikilohydric (Raven 1985; Watkins and Cardelús 2012; Krieg et al. 2019) and utilize ecophysiological strategies distinct from most sporophytes. Gametophytes are in a perpetual state of equilibrium with their surrounding environment. The spatial, structural, and nutritional independence of these life stages has been hypothesized to increase the potential arsenal of modifications to reproduction and/or physiology that can impact niche occupancy and/or geographic distribution (Krieg and Chambers 2022). However, the complex interactions of gametophyte and sporophyte generations is often overlooked when botanists explore the climate niche occupancy and/or geographic distribution of ferns and lycophytes.

The last two decades have seen a keen interest in gametophyte ecophysiology. We now know that fern gametophytes show adaptive acclimation to growth conditions, including light (Johnson et al. 2000; Fernández‐Marín et al. 2012), salt stress (Li and Ong 1998; Pangua et al. 2009), and nutrient availability (DeSoto et al. 2008; Goodnoe and Hill 2016). In particular, multiple studies have shown that fern gametophytes of some species demonstrate remarkable stress tolerance by entering dormancy for extended periods until later resuming metabolic function (Ong and Ng 1998; Watkins Jr., Mack, Sinclair, and Mulkey 2007; Chambers et al. 2017). Despite the growth in interest in fern gametophyte physiological function, only a few studies have made connections between gametophyte physiology and aspects of their ecology and/or distribution (Brock et al. 2019; Krieg and Chambers 2022). For example, epiphytic and terrestrial gametophytes in tropical habitats exhibit adaptive differences in morphology and desiccation tolerance (Watkins Jr., Mack, Sinclair, and Mulkey 2007; Watkins Jr., Mack, and Mulkey 2007). Chambers et al. (2017) found that populations of *Vittaria appalachiana* (a species that is only known in the gametophyte stage) can exhibit physiological adaptation to temperature that differs across its range, even when gametophyte populations occurred in climatically buffered microclimate locations such as rock shelters. While the role of physiological function in shaping geographic distributions and climate niche occupancy has been extensively studied in vascular plant sporophytes (Maire et al. 2015; Yang et al. 2019; Joswig et al. 2022; Smith et al. 2023), including some fern sporophytes (Hietz and Briones 1998; Saldaña et al. 2005, 2007; Watts and Watkins 2021), the extent to which fern gametophytes exhibit physiologies and distributions that differ from their sporophytic counterpart has received considerably less attention. Characterizing fern ecophysiological diversity in the gametophyte life stage is critical to forming a more holistic view of spatial patterns of plant biodiversity and ferns in particular.

Given the long‐distance dispersal potential (by wind and global air currents) exhibited by fern spores, the abundance of fern spores produced by an individual plant, and the ability of some species to establish persistent gametophyte‐only populations (Dassler and Farrar 2001; Pinson et al. 2017), geographic range expansion by fern gametophytes may be underpinned by adaptive physiological variation. Indeed, gametophytes of some species have been observed outside the geographic distribution of their conspecific sporophytes (Rumsey et al. 1998; Ebihara et al. 2013; Duffy et al. 2015; Kuo et al. 2017; Lee et al. 2020; Park et al. 2020; Pinson et al. 2022; Wu et al. 2022). Recent advances in genetic sequencing (e.g., tissue‐direct PCR and DNA barcoding) have greatly improved the feasibility of including fern gametophytes in field surveys (Nitta et al. 2017; Nitta and Chambers 2022; Quinlan et al. 2022), providing more gametophyte geographic occurrence data than ever before. Although recent advances have catalyzed the collection of crucial locality data, a methodological framework has not been developed to examine the prevalence and strength of ecological coordination between these independent life stages.

From deserts to cloud forests, ferns exhibit incredible ecological diversity (Mehltreter et al. 2010) and are the second‐largest group of vascular land plants (PPG I 2016). Quantifying the ecological coordination between fern gametophyte and sporophyte life stages and the factors that may promote or constrain such coordination has the potential to reveal novel mechanisms that generate spatial patterns of plant biodiversity. Here, we suggest how integrating physiological, ecological, and environmental data can help address three outstanding questions in fern ecology and biodiversity: (*Q1*) Is there evidence for the expansion of geographic and climate occupancy by fern gametophytes beyond their conspecific sporophytes? (*Q2*) Does gametophyte climate niche expansion occur randomly across environments or along specific axes of climate variation? (*Q3*) Is there evidence for ecophysiological adaptation across specific axes of climate variation in fern gametophytes that may promote and constrain the potential for gametophyte establishment?

Given the presence of broad and passive spore dispersal in ferns and theoretical and empirical evidence that physiological function is a primary driver of species distributions, we hypothesized that fern gametophytes could extend beyond the geographic range of sporophytes into environments that sporophytes do not currently occupy and that gametophytes exhibit physiological differences across broad environmental gradients. To test for a generational separation of niche space, we compared the geographic and climatic occupancy of conspecific gametophyte and sporophyte generations (*Q1* and *Q2*). To test for evidence of gametophyte ecophysiological adaptation and trade‐offs, we analyzed trends in gametophyte physiology across broad environmental gradients (*Q3*). Finally, we synthesize our findings to propose a potential role of environmental factors on physiological mechanisms that may promote or constrain the spatial and ecological patterns of fern biodiversity.

## 2 Materials and Methods

### 2.1 Constructing a Fern Gametophyte Occurrence Database

To compile a gametophyte occurrence dataset, we gathered and curated a list of 11 published studies on gametophyte distribution and/or ecology, supplemented with a Google Scholar literature search using the search terms: “independent gametophyte” OR “separation of generations” OR “intergenerational ecological niche separation” OR “sporophyte‐less” (File S1). We recorded geographic locations from published studies where gametophytes were observed, studied, or collected. We georeferenced published maps from that list of publications and extracted point occurrences in QGIS (QGIS.org, 2023). This search procedure resulted in 23 species of ferns with geographic coordinates of gametophytes that span diverse ecological habitats and three plant orders (Marattiales, Hymenophyllales, and Polypodiales) (Table S2). Across all gametophyte occurrences derived from the literature, the presence or absence of sporophytes was also recorded. In addition to occurrence data for sporophytes reported in publications, we queried the Global Biodiversity Information Facility (GBIF; www.gbif.org), Atlas of Living Australia (ALA; www.ala.org.au) and Integrated Digitized Biocollections (iDigBio; www.idigbio.org) databases for sporophyte occurrence records using the *spocc* R package (Chamberlain et al. 2016) and *rgbif* R package (Chamberlain et al. 2020). A complete species list with observation counts and citation information for comparing geographic and environmental occupancy between gametophytes and sporophytes within species can be found in File S1, including direct links to GBIF data downloads.

### 2.2 Compiling Environmental Data

To compile a dataset of contemporary global environmental conditions, we obtained global climate rasters from CHELSA (Karger et al. 2021), TerraClimate (Abatzoglou et al. 2018), and SoilTemp (Lembrechts et al. 2022). All environmental data were obtained at ~1km^2^ spatial resolution, and environmental values were extracted at each occurrence point in QGIS using the *point‐sampling* tool QGIS (QGIS.org, 2023). The ‘biovariables’ function in the *climatica* R package was used to calculate 86 variables derived from monthly data for precipitation, air temperature, potential evapotranspiration, vapor pressure deficit, soil temperature, and soil moisture content (see File S2 for a complete list).

Geographic occurrence records gathered directly from published papers were validated to occur on land and checked for duplicate records of the same species and life stage. Geographic occurrence records from databases were cleaned in a multistep procedure. First, only records with preserved specimens were retained. Next, the R package CoordinateCleaner was used to remove records that were likely not representing the wild distribution of the species (Zizka et al. 2019). Then, duplicate records were removed, and the remaining coordinate records were thinned to ~1 km^2^ to reduce sampling bias. Third, Isolation Forests were used for each species to identify potential environmental outliers using the *solitude* R package (Liu et al. 2012), and records with an anomaly score greater than 0.75 were removed (with 0 indicating a point that is difficult to isolate from the group and 1 indicating a point that is very easy to isolate from the group) (Liu et al. 2008, 2012). Finally, coordinates were plotted in QGIS and manually checked for poor quality points based on habitat and locality descriptions from the Flora of North America (http://www.efloras.org/) and the KEW World List of Plants (2023).

### 2.3 Developing Metrics of Geographic and Climate Niche Differences Between Life Stages

To quantify the potential extent to which gametophytes occupy broader geographic distributions than sporophytes (see Questions 1 and 2), we formulated a metric of geographic expansion potential (GEP) enabled by the gametophyte generation such that

(1) $$\mathrm{GEP}=G_{\mathrm{geo}}/\left(G_{\mathrm{geo}}+S_{\mathrm{geo}}\right)$$

where *G*_geo_ is the geographic area occupied by gametophytes only (i.e., no sporophytes have been observed), and *S*_geo_ is the geographic area occupied by sporophytes. We assume that observations of sporophytes also indicate the recent presence of a gametophyte. A GEP of 1 indicates that all geographic occurrence records of a species are of gametophytes only. A GEP of 0 indicates that the gametophyte geographic area only occurs within the geographic area occupied by sporophytes.

To quantify the extent to which gametophytes occupy broader climate space than sporophytes (see Questions 1 and 2), we formulated an analogous metric of climate expansion potential (CEP) enabled by the gametophyte generation such that

(2) $$\mathrm{CEP}=G_{\mathrm{env}}/\left(G_{\mathrm{env}}+S_{\mathrm{env}}\right)$$

where *G*_env_ is the climate occupied by gametophytes only (i.e., no sporophytes have been observed), and *S*_env_ is the climate occupied by sporophytes. We assume that observations of sporophytes also indicate the presence of a gametophyte. A CEP of 1 indicates that the climate occupancy of a species (gametophyte and sporophyte generations) is represented by gametophytes only. A CEP of 0 indicates that the gametophyte climate niche occurs within the climate niche occupied by sporophytes. We multiplied CEP and GEP by 100 to represent each as a percentage. Note that this metric of climate expansion (CEP) is sensitive to the spatial scale of the climate data. Ideally, climate data would come from the exact location of the plant at the microclimate scale, however, those data do not exist, and we take multiple steps to make the global rasters that we used more applicable (see below and Section 4).

### 2.4 Characterizing Geographic and Climate Niche Occupancy of Fern Life Stages

To quantify the geographic expansion potential (GEP) of gametophytes (see Questions 1 and 2), we estimated the geographic area range of each life stage within each species. We used point buffers with a 1 km radius around each geographic occurrence record using the *rgeos* R package (Bivand et al. 2019) and partitioned the geographic area of each species into gametophyte only and sporophyte and gametophyte areas, accounting for their overlap (see Equations 1 and 2).

To quantify the climate expansion potential (CEP; see Equation 2, see Questions 1 and 2) of gametophytes, we characterized the environmental occupancy of each life stage within each species using four main approaches. First, the CEP values were calculated individually for each of the 86 environmental variables, and the overall mean across variables was calculated for each species. In three other approaches, we constructed Gaussian hypervolumes using the “hypervolume_gaussian” function, multi‐dimensional polytopes (i.e., convex hulls in many dimensions) using the “expectation_convex” function, and hyperballs using the “expectation_ball” function in the *hypervolume* R package (Blonder et al. 2018). These four approaches all produced strong linear relationships between GEP and CEP, and estimated some species to have a positive GEP but a CEP of zero (e.g., Figure 2). However, we found that the probabilistic nature of some of the approaches (e.g., gaussian hypervolumes) produced non‐zero estimations of “sporophyte CEP” (i.e., the estimated sporophyte niche is larger than the niche estimated with sporophytes and gametophytes), which is not possible given that all sporophyte occurrences were also assumed to indicate the presence of a gametophyte. The percentage of a species' climate occupancy erroneously attributed to sporophytes‐only was smallest using the “expectation_convex” function, which ranged from 0% to 4%; therefore, we used multi‐dimensional polytopes to estimate climate occupancy throughout our analyses. In addition, we subtracted any non‐zero estimate of a ‘sporophyte CEP’ to conservatively estimate gametophyte CEP (Equation 2). All erroneous estimations of ‘sporophyte CEP’ and the conservatively estimated gametophyte CEP are reported in File S4.

To detect whether gametophytes may occupy novel climatic space along specific axes of climate variation (see Question 2), we estimated a CEP based on temperature variables only (CEP_Temperature_; e.g., mean annual temperature, temperature of wettest quarter, mean annual soil temperature, etc.), water variables only (CEP_Water_; e.g., mean annual precipitation, precipitation of coldest quarter, mean annual soil water content, etc.), and environmental factors driven by their interdependence (e.g., potential evapotranspiration, aridity, vapor pressure deficit, etc.; CEP_Temperature×Water_). Note that CEP_Temperature×Water_ represents specific climate variables and does not indicate a statistical interaction. To better account for differences between the epiphytic and terrestrial climate occupancy, we included soil temperature and soil moisture variables for terrestrial species but not for epiphytic species. Before characterizing environmental occupancy, we performed principal component analyses for each species to reduce the number of dimensions and make the n‐dimensional analyses of environmental occupancy more computationally tractable. For each species and type of CEP, we used the number of principal component axes that most closely captured 80% of the cumulative variation, which generally ranged from 1 to 4 axes. A complete list of variables, source information, and their grouping in analyses can be found in Files S2 and S4.

To investigate the coordination between the degree of geographic and environmental separation between gametophytes and sporophytes (see Questions 1 and 2), we performed phylogenetic reduced major axis regressions using the “phyl.RMA” function in the *phytools* R package (Revell 2012). We report Pagel's lambda (*λ*), which represents the degree to which trait variation aligns with the topology of a phylogenetic tree and is used as a statistical adjustment to the covariance matrix among species with shared ancestry (Pagel 1999; Pearse et al. 2023). We obtained a phylogeny by pruning the fern tree of life available in the *ftolr* R package v. 1.6.0 (Nitta et al. 2022). Data points that exhibit positive GEP and a CEP equal to zero were excluded from regression analyses. Importantly, we did not analyze the absolute values of CEP or GEP against other types of data because they are sensitive to the number of occurrence points (and how well they represent the realized niche) of gametophytes and sporophytes, and gametophyte occurrence data are relatively scarce. Instead, we focused on testing the coordination of CEP and GEP (slope of relationship), which is less sensitive to the number of occurrence points and only assumes that occurrence records represent a random sample of the realized niche.

### 2.5 Harmonizing Ecophysiological Data of Fern Gametophytes

To test if fern gametophytes show evidence of climate preference and ecophysiological adaptation across environmental gradients (see Question 3), we gathered ecophysiological data from the literature for gametophytes of 18 fern species that represent broad phylogenetic, ecological, and biogeographical diversity. These 18 species span four plant orders (Osmundales, Cyatheales, Hymenophyllales, and Polypodiales) and 5 biomes across 6 continents. Four species are epiphytic, and 14 species are terrestrial.

Ecophysiological data were gathered from published sources, focusing on traits related to photosynthesis and water relations. We found gametophyte photosynthetic data for 10 species and dehydration responses for 13 species (with 5 species that have both photosynthetic and desiccation resistance data, for a total of 18 unique species with any data). Data were gathered on the desiccation rate from Watkins Jr., Mack, Sinclair, and Mulkey (2007) and Pittermann et al. (2013). Data were gathered on the maximum rates of photosynthesis from Farrar et al. (2008) and full light response curves for five additional species (Friend 1974; Hagar and Freeberg 1980; Li and Ong 1998; Johnson et al. 2000). We recorded data from published tables or extracted from figures using WebPlotDigitizer (v.4.4, https://apps.automeris.io/wpd/).

We took several steps to increase the comparability of published data. For example, spot measurements of photosynthetic rates were most often recorded at 100 μmol m^−2^ s^−1^ PAR, and light response curves sometimes exceeded 100 μmol m^−2^ s^−1^ PAR. To harmonize the photosynthetic data, we extracted the photosynthetic rate achieved at 100 μmol m^−2^ s^−1^ PAR from light response curves using the *photosynthesis* R package (Stinziano et al. 2021), and used these light standardized rates throughout the analyses. In addition, some published data on fern gametophyte physiology were originally measured across experimental treatments, in which case, we extracted data from experimental controls (e.g., Li and Ong 1998). Most studies reported gametophyte photosynthetic rates in units of μmol CO_2_ g^−1^ s^−1^. However, Johnson et al. (2000) examined light responses in gametophytes of *Vandenboschia speciosa* (Willd.) G. Kunkel per gram of chlorophyll and did not report the amount of chlorophyll per dry weight. To convert these data to rates per unit dry mass, we used the average reported values of gametophyte chlorophyll per dry mass in Li and Ong (1998). In other cases, units of gametophyte gas‐exchange were reported in units of O_2_ production, and we converted O_2_ production to CO_2_ assimilation (Kaplan and Björkman 1980; Delieu and Walker 1981; Hall and Rao 1999).

In Watkins Jr., Mack, Sinclair, and Mulkey (2007), gametophytes of 12 species, including *Pityrogramma calomelanos* , were held at a relative humidity of 50% (−94.57 MPa) for 24 h, and the rate of water loss was expressed as change in relative water content (ΔRWC min^−1^). Pittermann et al. (2013) also measured the rate of gametophyte water loss expressed as relative water content (ΔRWC min^−1^) for two species, *Pityrogramma calomelanos* and *Campyloneurum brevifolium*, but did not report the atmospheric water potential. Because at a given tissue conductance from the gametophyte to the atmosphere, the rate of water loss is approximately linearly related to the atmospheric water potential, we compared the two different rates of water loss for *Pityrogramma calomelanos* presented in each study to derive a linear conversion factor of 0.646. We applied this conversion factor to the rate of water loss for *Campyloneurum brevifolium* (−1.54 ΔRWC min^−1^) gathered from Pittermann et al. (2013) to yield an estimated rate of water loss of −0.998 (ΔRWC min^−1^) if it were measured in a similar atmospheric water potential as in Watkins Jr., Mack, Sinclair, and Mulkey (2007). A complete list of species, ecophysiological traits, and their published sources can be found in File S3.

### 2.6 Global Environmental Drivers of Gametophyte Ecophysiology

To test for evidence of global convergence in gametophyte physiological function (see Question 3), we used phylogenetic linear regressions using the *phylolm* R package (Ho et al. 2016) and the pruned phylogeny described above (Nitta et al. 2022). To help account for differences in environmental conditions experienced by terrestrial and epiphytic species in trait × environment analyses, we synthesized metrics of temperature and water derived from air and soil, depending on whether gametophytes are terrestrial or epiphytic. For example, the mean annual temperature experienced by gametophytes was synthesized using mean annual air temperature (CHELSA; Karger et al. 2021) for epiphytic gametophytes and mean annual soil temperature (Lembrechts et al. 2022) for terrestrial gametophytes. Metrics of water inputs were similarly synthesized using precipitation (CHELSA; Karger et al. 2021) for epiphytic gametophytes and soil water content (TerraClimate; Abatzoglou et al. 2018) for terrestrial gametophytes. In all cases of synthesized variables, input variables shared the same units or were converted to the same units. If environmental parameters were not available at the soil surface level, then parameters modeled at 2 m height in the air were used. A complete list of variables, source information, and their grouping in analyses can be found in File S2.

**FIGURE 1:**
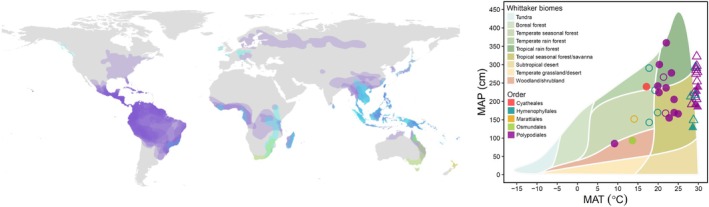
Geographic and climate occupancy of fern species in this study. Left: A global map of terrestrial Earth with generalized distributions of each of the 40 species included in this study. Right: All points are the mean sporophyte values for mean annual precipitation (MAP) and either mean annual air temperature for epiphytes or soil temperature for terrestrial species (MAT). Open points indicate species for which we have confirmed gametophyte occurrence records, and filled points indicate species for which we have gametophyte ecophysiological data. Triangles indicate epiphytes, and circles represent terrestrial species. Geographic ranges (left) and climate occupancy points (right) collectively represent 40 fern species across five plant orders (across both panels: Cyatheales, red; Hymenophyllales, teal; Marattiales, yellow; Osmundales, green; Polypodiales, purple).

To test whether growth habit (epiphytic vs. terrestrial; see Table S1) was a significant predictor of trait × environment outcomes, we added growth habit as a fixed factor to the phylogenetic linear models. No trait × environment model identified growth habit as a significant factor (data not shown), thus the simpler regression model was used, pooling data from both growth habitats (Figure 1).

## 3 Results

### 3.1 Gametophytes Exhibit Distinct Patterns of Climate Niche Expansion

**FIGURE 2:**
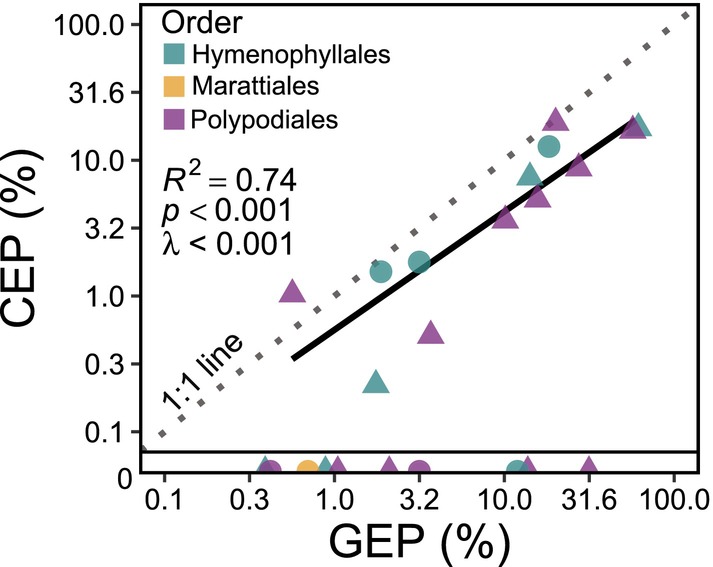
Gametophyte geographic separation, climate separation, and their coordination. Linear relationship between gametophyte climate expansion potential (CEP) and geographic expansion potential (GEP) (*R* ^2^ = 0.74, *p* < 0.001, *λ* < 0.001) for thirteen fern species. Triangles are epiphytes and circles are terrestrial species. Data represent 23 fern species across three plant orders (Hymenophyllales, teal; Marattiales, yellow; Polypodiales, purple) that exhibit GEP and/or CEP (see Equations 1 and 2). Ten data points along the *x*‐axis below the horizontal line are species that exhibit positive GEP and a CEP equal to zero.

**FIGURE 3:**
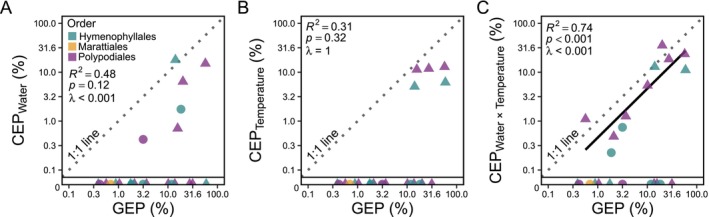
Coordination of gametophyte geographic expansion (GEP) and climate expansion (CEP) along three primary axes of climate variation on log‐scale. (A) no relationship between gametophyte GEP and CEP derived from water variables only (e.g., precipitation and soil water content; CEP_Water_), (B) no relationship between gametophyte GEP and CEP derived from temperature variables only (e.g., air and soil temperatures; CEP_Temperature_), (C) linear relationship between gametophyte GEP and CEP derived from variables strongly dependent on the interaction between temperature and water (e.g., potential evapotranspiration, vapor pressure deficit; CEP_Water×Temperature_). Triangles are epiphytes and circles are terrestrial species. Data represent 23 fern species across three plant orders (Hymenophyllales, teal; Marattiales, yellow; Polypodiales, purple) that exhibit GEP and/or CEP (see Equations 1 and 2). Data points along the *x*‐axis below the horizontal line are species that exhibit positive GEP and a CEP equal to zero.

Phylogenetic regressions revealed that the degree of geographic expansion potential (GEP) exhibited by a fern species is significantly correlated with their climate expansion potential (CEP) for 13 of 23 species (*R* ^2^ = 0.74, *p* < 0.001, *λ* < 0.001; Figure 2), with 10 species exhibiting geographic expansion but occupying the same climate space as their sporophytes. Performing phylogenetic regressions on species' CEP independently for temperature, water‐related variables, and variables corresponding to their interdependence (e.g., PET, VPD, etc.) revealed that the coordination between gametophyte geographic expansion occurs along specific axes of climate variation. Gametophyte GEP was not significantly correlated with CEP_Water_ (*R* ^2^ = 0.48, *p* < 0.12, *λ* < 0.001; Figure 3A) or CEP_Temperature_ (*R* ^2^ = 0.31, *p* < 0.32, *λ* = 1; Figure 3B) with only 6 and 5 species, respectively, showing any degree of novel climate expansion. In contrast, we found strong coordination between the degree of gametophyte geographic expansion and their climate expansion along axes driven by the interaction between temperature and water (CEP_Temperature×Water_) for 11 of 23 species (*R* ^2^ = 0.74, *p* < 0.001, *λ* < 0.001; Figure 3C), with 12 species exhibiting geographic expansion but occupying climate space similar to their sporophytes.

### 3.2 Gametophytes Show Convergence in Ecophysiological Functioning

**FIGURE 4:**
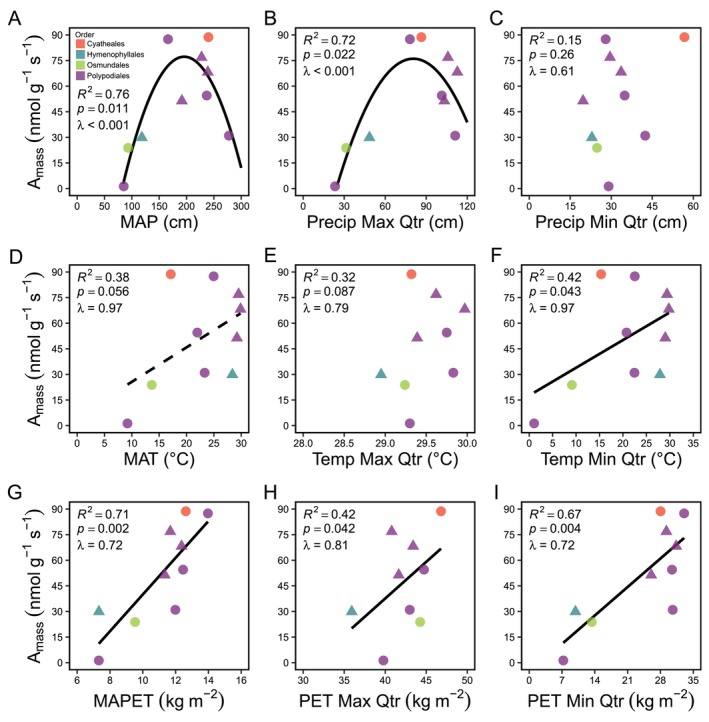
Environmental drivers of gametophyte photosynthetic function. Relationships between gametophyte photosynthetic rate per mass (*A*_mass_) and (A) mean annual precipitation (MAP), (B) mean precipitation of the quarter with the highest precipitation (for epiphytes) or soil moisture (for terrestrial gametophytes) (Precip Max Qtr), (C) mean precipitation of the quarter with the least precipitation (for epiphytes) or soil moisture (for terrestrial gametophytes) (Precip Min Qtr), (D) mean annual air temperature (for epiphytes) or soil temperature (for terrestrial gametophytes) (MAT), (E) mean temperature of the quarter with the highest air temperature (for epiphytes) or soil temperature (for terrestrial species) (Temp Max Qtr), (F) mean temperature of the quarter with the lowest air temperature (for epiphytes) or soil temperature (for terrestrial gametophytes) (Temp Min Qtr), (G) mean annual potential evapotranspiration (MAPET), (H) mean potential evapotranspiration of the quarter with the highest potential evapotranspiration (PET Max Qtr), and (I) mean potential evapotranspiration of the quarter with the lowest potential evapotranspiration (PET Min Qtr). Triangles are epiphytes and circles are terrestrial species. Data represent 10 fern species across four plant orders (Cyatheales, red; Hymenophyllales, teal; Osmundales, green; Polypodiales, purple).

Using data from 10 fern species across four plant orders, 5 biomes, and 6 continents, we recovered evidence of convergence in fern gametophyte photosynthetic physiology across global environmental gradients. Phylogenetic regressions showed that gametophyte photosynthetic rate per mass (*A*_mass_) is significantly and monotonically correlated with mean annual precipitation (MAP) (*R* ^2^ = 0.76, *p* = 0.011, *λ* < 0.001; Figure 4A) and mean precipitation of the quarter with the highest precipitation (for epiphytes) or soil water content (for terrestrial gametophytes) (*R* ^2^ = 0.72, *p* = 0.022, *λ* < 0.001; Figure 4B), but is not correlated with mean precipitation of the quarter with the lowest precipitation (for epiphytes) or soil water content (for terrestrial gametophytes) (*R* ^2^ = 0.15, *p* = 0.026, *λ* = 0.84; Figure 4C). Gametophyte *A*_mass_ was marginally significantly correlated with mean annual temperature (MAT) (*R* ^2^ = 0.38, *p* = 0.056, *λ* = 0.97; Figure 4D) but not with mean temperature of the quarter with the highest air temperature (for epiphytes) or soil temperature (for terrestrial gametophytes) (*R* ^2^ = 0.32, *p* = 0.087, *λ* = 0.79; Figure 4E). Gametophyte *A*_mass_ was positively correlated with mean temperature of the quarter with the lowest air temperature (for epiphytes) or soil temperature (for terrestrial gametophytes) (*R* ^2^ = 0.42, *p* = 0.043, *λ* = 0.97; Figure 4F). Gametophyte *A*_mass_ was significantly positively correlated with mean annual potential evapotranspiration (MAPET) (*R* ^2^ = 0.71, *p* = 0.002, *λ* = 0.72; Figure 4G), mean potential evapotranspiration of the quarter with the highest potential evapotranspiration (PET Max Qtr) (*R* ^2^ = 0.42, *p* = 0.042, *λ* = 0.81; Figure 4H), as well as mean potential evapotranspiration of the quarter with the lowest potential evapotranspiration (PET Min Qtr) (*R* ^2^ = 0.67, *p* = 0.004, *λ* = 0.72; Figure 4I).

**FIGURE 5:**
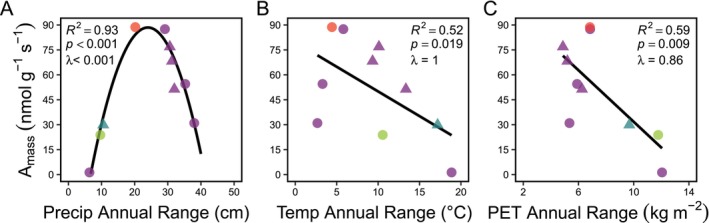
Annual climate stability constrains gametophyte photosynthetic function. Relationships between gametophyte photosynthetic rate per mass (*A*_mass_) and the annual range of (A) precipitation (Precip Annual Range), (B) temperature (Temp Annual Range), and (C) potential evapotranspiration (PET Annual Range). Triangles are epiphytes and circles are terrestrial species. Data represent 10 fern species across four plant orders (Cyatheales, red; Hymenophyllales, teal; Osmundales, green; Polypodiales, purple).

We recovered significant relationships between gametophyte photosynthetic function (*A*_mass_) and the annual variability of climate. For example, gametophyte *A*_mass_ exhibited a non‐linear, monotonic relationship with annual range of precipitation (*R* ^2^ = 0.93, *p* < 0.001, *λ* < 0.001; Figure 5A), and a negative relationship with the annual range of temperature (*R* ^2^ = 0.52, *p* = 0.019, *λ* = 1; Figure 5B) and annual range of potential evapotranspiration (*R* ^2^ = 0.59, *p* = 0.009, *λ* = 0.86; Figure 5C).

**FIGURE 6:**
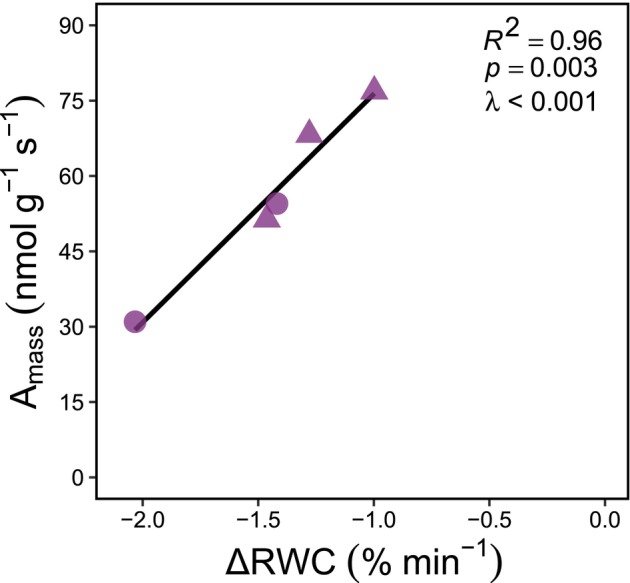
Gametophyte photosynthetic rate and desiccation resistance jointly determine gametophyte ecophysiological functioning. The linear relationship between gametophyte photosynthetic rate per mass (*A*_mass_; *x*‐axis) and the rate at which gametophytes resist desiccation (ΔRWC; *y*‐axis). Triangles are epiphytes and circles are terrestrial species. Data represent five fern species across five genera, two families, and one plant order (Polypodiales, purple).

We also discovered a very strong positive relationship between gametophyte *A*_mass_ and gametophyte desiccation resistance (ΔRWC) (*R* ^2^ = 0.96, *p* < 0.003, *λ* < 0.001; Figure 6).

## 4 Discussion

Understanding the eco‐evolutionary factors that drive species' ecological distributions is one of the most important questions in biodiversity research (von Humboldt and Bonpland 1805; Darwin 1871; Gilpin 1979; Sexton et al. 2009; Sutherland et al. 2013). Several studies have documented the presence of independent fern gametophyte populations outside the geographic range of their corresponding sporophyte (Rumsey et al. 1998; Ebihara et al. 2013; Duffy et al. 2015; Kuo et al. 2017; Lee et al. 2020; Park et al. 2020; Pinson et al. 2022; Wu et al. 2022), yet whether the geographic separation between life stages represents climatic separation driven by physiological separation is poorly understood. Tobler's first law of geography concerning spatial autocorrelation states that locations closer to each other will be more similar than locations that are further away (Waters 2018). Thus, if fern spore dispersal is largely random in direction and distance and gametophytes establish, then we would expect the geographic distance and climatic similarity to correlate across all climate variation axes simply due to spatial autocorrelation (but see Gómez‐Noguez et al. 2022; Brock 2025 on the role of wind and spore size). Indeed, we found strong evidence for the coordination of geographic and environmental separation between gametophytes and sporophytes (Q1; Figure 2). However, when we decomposed gametophyte climate niche exploration into the underlying components, we also discovered that fern gametophytes occupy novel climatic space along specific axes of environmental variation related to the interdependence between water availability and temperature (e.g., VPD, PET; CEP_Temperature×Water_) (Q2; Figure 3). The evidence of novel climate expansion along specific axes contrasts with expectations of random gametophyte climate occurrence and suggests the presence of ecophysiological mechanisms that govern the coordination between fern gametophyte and sporophyte geographic and environmental occupancy. Importantly, the lack of evidence for gametophyte expansion along axes related to water (precipitation, soil water content, etc.) is further evidence that having motile sperm for aqueous fertilization likely does not constrain fern distributions (Watkins Jr., Mack, Sinclair, and Mulkey 2007; Farrar et al. 2008; Haufler et al. 2016). Identifying the axes of environmental variation along which fern gametophytes occupy novel climatic space relative to their sporophytes opens new areas of research to determine the mechanisms underlying spatial and temporal patterns of fern biodiversity.

Our discovery of novel niche expansion suggests that gametophyte niche expansion may be driven by physiological mechanisms, in accordance with theory that a species' fundamental niche is driven by its ecophysiological function and stress tolerances (Hutchinson 1957; Kearney et al. 2010; Kraft et al. 2015; Sperry et al. 2017; Smith et al. 2023). When we examined variation in gametophyte photosynthesis, we found strong correlations with photosynthetic rate per mass (*A*_mass_) and native climate conditions related to precipitation, temperature, and atmospheric water demand (i.e., potential evapotranspiration). In particular, we found strong linear relationships with mean annual potential evapotranspiration (MAPET) (Q3; Figure 4G–I). The strength and consistency of these relationships mirror those recovered in our analyses of gametophyte GEP and CEP_Temperature×Water_ (Figure 3C) and are consistent with physiological adaptation being a primary mechanism by which gametophytes occupy novel climatic space (Figures 2, 3, 4, 5, 6). Importantly, the niche expansion and physiological data are largely independent datasets with only one overlapping species (*Vandenboschia speciosa*) (Figure 1). Their congruence supports the potential generality of our findings across ferns. Indeed, several studies have also shown that gametophyte desiccation response to atmospheric water demand (i.e., PET, VPD, etc.) is a primary axis of physiological adaptation in ferns (Watkins Jr., Mack, Sinclair, and Mulkey 2007; Chambers et al. 2017; Nitta et al. 2021). Although our data are consistent with physiological mechanisms driving the climate niche separation observed between fern gametophytes and sporophytes, we cannot rule out the potential impact of biotic factors. For example, it is possible that gametophytes and sporophytes both possess the physiological adaptation to establish in the same climatic niche space, and that biotic interactions limit the geographic occupancy of sporophytes more than gametophytes through mechanisms like herbivory (Mesipuu et al. 2009; Castrejón‐Varela et al. 2022; Tessier 2025).

Comparing physiological differences between sporophytes and gametophytes could improve our understanding of niche separation between life stages in ferns; however, very few studies have compared the physiology of fern gametophytes to their conspecific sporophytes. Nitta et al. (2021) conducted surveys of photochemistry and desiccation responses in gametophytes and sporophytes of several species in Hymenophyllales from Moorea (French Polynesia). The authors found that the degree of recovery from desiccation was correlated with the native site VPD in sporophytes but not in gametophytes. The lack of relationship between gametophyte physiology and native climate reported in Nitta et al. (2021) could be due to the small spatial scale of the study on the island of Mo'orea if macroclimate variation is small and microclimate variation is large across the study area. Our discovery of a broad convergence in environmentally driven gametophyte physiological function may be due to the inclusion of a global set of ecologically and phylogenetically diverse fern species in our analyses and points to an untapped potential to investigate the physiological mechanisms that drive broad spatial patterns of fern biodiversity.

**FIGURE 7:**
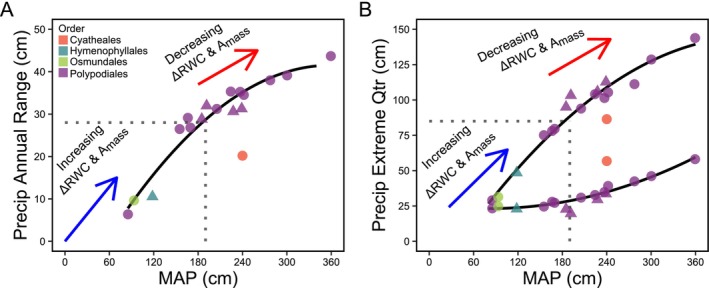
Mean annual precipitation is correlated with annual range in precipitation and both are principally driven by precipitation maximums. Relationships between mean annual precipitation (MAP) and (A) the annual range of precipitation (Precip Annual Range), and (B) quarterly extremes in precipitation (Precip Extreme Qtr). In panel B, the upper line indicates the mean precipitation of the quarter with the highest precipitation (for epiphytes) or soil moisture (for terrestrial gametophytes) and the lower line indicates the mean precipitation of the quarter with the least precipitation (for epiphytes) or soil moisture (for terrestrial gametophytes). In both panels, arrows indicate the direction of physiological functions in the positive (blue arrows) and negative (red arrows) direction. Triangles are epiphytes and circles are terrestrial species. Points represent 18 fern species with ecophysiological data across four plant orders (Cyatheales, red; Hymenophyllales, teal; Osmundales, green; Polypodiales, purple).

The strong convergence in gametophyte ecophysiological adaptation across broad environmental scales (Figures 4 and 6) may help enable novel niche expansion in some fern gametophytes, however, we also discovered evidence of physiological and environmental factors that may constrain the ecology and distribution of ferns. For example, gametophyte photosynthetic performance (*A*_mass_) exhibited a steep initial increase, an apparent peak, and a steep decrease with respect to mean annual precipitation and the amount of precipitation in the wettest quarter (Figure 4). These trends across diverse fern lineages suggest the presence of an environmental threshold, across which distinct selective pressures are imposed on gametophyte physiological function. However, the potential impact of variation in fern gametophyte photosynthetic rate on survival and fitness across environmental gradients has yet to be described. By combining data on photosynthesis and water relations, we discovered that gametophyte photosynthetic performance (*A*_mass_) was strongly positively correlated with desiccation resistance (ΔRWC) (Figure 6), which also explains the strong linear relationship with mean annual potential evapotranspiration (MAPET) (Figure 4G–I). We further examined the covariation of environmental factors for species in our dataset to characterize this potential environmental threshold better. We found that mean annual precipitation was strongly correlated with the annual range in precipitation, and that variation in the annual range of precipitation was principally driven by changes in the precipitation of the wettest quarter (Figure 7). This discovery suggests that selective pressures may act to increase desiccation resistance via increased photosynthetic rates as water availability becomes more variable, up to a threshold where gametophytes receive enough water to relax selection on desiccation resistance due to increased quarterly precipitation maximums (Figure 7). At this threshold, gametophyte ecophysiological function may become limited by other resources (light and/or nutrients), which implicates annual and/or seasonal fluctuations in the environment as key drivers of the establishment and growth of gametophyte populations.

## 5 Future Directions

The broad macroevolutionary pattern from bryophytes to flowering plants of shifting away from an independent gametophyte has led many to believe that the continued reliance on an independent gametophyte represents a major evolutionary constraint in plant evolution. However, our data suggest that in the case of seed‐free vascular plants like ferns, the independent gametophyte may be a principal reason for their ecological success. In this paper, we present evidence that fern gametophytes can occupy novel climate niche space relative to their sporophytes (Q1; Figure 2) and that this expansion occurs along specific axes of climate variation (Q2; Figure 3). However, our analyses also reveal that many species exhibit high geographic expansion (high GEP) but do not inhabit novel climates (CEP of zero), indicating that gametophytes of some species disperse to track favorable sporophyte climates while gametophytes of other species occupy novel climates. This suggests there may be a division of eco‐evolutionary strategy with respect to the coordination between sporophyte and gametophyte ecologies. Future research should elucidate the physiological, ecological, and evolutionary mechanisms that jointly comprise these distinct strategies of coordination. Future research should also consider additional factors that could contribute to this pattern, including biotic factors such as herbivory on sporophytes but not gametophytes, and/or temporal lags between when gametophytes were observed and a potential unobserved sporophyte that could arise later. We also discovered evidence for broad convergence in gametophyte photosynthetic performance (*A*_mass_) driven by environmental gradients (Figures 4 and 6), and that environmental maxima and variability may interact to impose distinct selective pressures on gametophyte ecophysiological function (Figures 6 and 7). However, both the mechanistic link between gametophyte photosynthetic performance (*A*_mass_) and desiccation resistance and their impact on fitness are unknown.

One limitation to understanding seed‐free plant biology is the relative scarcity of gametophyte occurrence data. For our study, the paucity of these data prohibited us from inferring the realized niche of gametophytes, and instead restricted our analyses to the relative coordination of CEP and GEP only. In addition, microclimate data for taxa of similar phylogenetic and ecological diversity to those analyzed here do not currently exist. By using coarse‐scale data for air and soil surfaces, our analyses rely on the assumption that the difference between the microclimate (unmeasured) and macroclimate (estimated by global raster) at a given geographic location is less than the difference between the microclimate at a given geographic location and the macroclimate at other locations across broad geographic and climatic scales. Future progress in spatial biodiversity research of seed‐free taxa will require a greater focus on recording gametophytes' geographic and (micro‐ and macro‐) climatic occupancy across ecologically and phylogenetically diverse species.

## Author Contributions

**Christopher P. Krieg:** conceptualization (lead), data curation (equal), formal analysis (equal), investigation (lead), methodology (equal), validation (equal), visualization (lead), writing – original draft (lead), writing – review and editing (equal). **Jacob L. Watts:** conceptualization (supporting), data curation (equal), formal analysis (equal), investigation (equal), methodology (supporting), validation (supporting), visualization (supporting), writing – original draft (supporting), writing – review and editing (equal). **Sally M. Chambers:** conceptualization (equal), data curation (supporting), investigation (supporting), methodology (supporting), writing – original draft (supporting), writing – review and editing (equal). **Ameya Baxi:** data curation (supporting), investigation (supporting), writing – review and editing (supporting). **Katherine A. McCulloh:** methodology (supporting), writing – review and editing (supporting).

## Funding

This work was supported by the US National Science Foundation Division of Biological Infrastructure DBI‐1907033 (C.P.K.); US National Science Foundation Division of Integrative Organismal Systems IOS‐2243970 (C.P.K., K.A.M.).

## Conflicts of Interest

The authors declare no conflicts of interest.

## Supporting information


**Table S1:** List of gametophyte and sporophyte occurrence records for each of the 23 species.
**Table S2:** A list of species in this study, their phylogenetic order, habit, morphology, presence of gemmae, and inclusion in the ecophysiology (ecophys) or niche expansion dataset (ftl), or both.

**File S1:** ece373324‐sup‐0002‐FileS1.csv.

**File S2:** ece373324‐sup‐0003‐FileS2.csv.

**File S3:** ece373324‐sup‐0004‐FileS3.csv.

**File S4:** ece373324‐sup‐0005‐FileS4.csv.

## Data Availability

All data are available in public repositories, the main text, and/or the Supporting Information. Data and code to reproduce figures were uploaded to Zenodo (November 17, 2023; https://doi.org/10.5281/zenodo.10150063).
