# Supplementary material for: Fern Gametophytes Exhibit Distinct Patterns of Niche Expansion and Convergence in Ecophysiological Functioning
Source: Ecol Evol. 2026 Apr 7;16(4):e73324. doi: 10.1002/ece3.73324 (PMC13055197; doi:10.1002/ece3.73324)
Supplement: Supplementary file 1 — Table S1: List of gametophyte and sporophyte occurrence records for each of the 23 species. Table S2: A list of species in this study, their phylogenetic order, habit, morphology, presence of gemmae, and inclusion in the ecophysiology (ecophys) or niche expansion dataset (ftl), or both. [file ECE3-16-e73324-s001.docx]

**Supporting Information**

Fern gametophytes exhibit distinct patterns of niche expansion

and convergence in ecophysiological functioning

Christopher P. Krieg^1^*, Jacob L. Watts^2^, Sally M. Chambers^3^,

Ameya Baxi^4^, Katherine A. McCulloh^4^

^1^ 1834 Wake Forest Rd, Department of Biology, Wake Forest University, Winston-Salem, NC,

USA

^2^ 1900 Pleasant St., Ecology and Evolutionary Biology Department; University of Colorado,

Boulder, CO, USA

^3^ 521 Lancaster Ave., Department of Biology, Eastern Kentucky University, Richmond, KY, USA
^4^ 430 Lincoln Dr., Department of Biology, University of Wisconsin, Madison, WI, USA

*correspondence: [kriegc@wfu.edu](mailto:christopher.p.krieg@gmail.com)

**Keywords**: climate, distribution, ecology, ecophysiology, ferns, gametophytes, niche

**This file includes**:

Tables: 2

**Table S1**. List of gametophyte and sporophyte occurrence records for each of the 23 species included in this study. See **File S1** for a complete list of citations for each species.

| **species name** | **gametophyte records** | **sporophyte records** |
| --- | --- | --- |
| *Abrodictyum dentatum* | 6 | 78 |
| *Adiantum trapeziforme* | 5 | 527 |
| *Antrophyum obovatum* | 7 | 202 |
| *Antrophyum reticulatum* | 5 | 127 |
| *Archigrammitis tahitensis* | 4 | 7 |
| *Arthropteris palisotii* | 4 | 453 |
| *Callistopteris apiifolia* | 19 | 214 |
| *Callistopteris baldwinii* | 4 | 14 |
| *Callistopteris baueriana* | 3 | 20 |
| *Calymmodon orientalis* | 5 | 5 |
| *Diplazium harpeodes* | 4 | 37 |
| *Hymenophyllum pallidum* | 3 | 290 |
| *Hymenophyllum wrightii* | 71 | 528 |
| *Lomagramma tahitensis* | 5 | 4 |
| *Lomariopsis boninensis* | 12 | 6 |
| *Lomariopsis brackenridgei* | 7 | 20 |
| *Loxogramme parksii* | 5 | 37 |
| *Pleurosoriopsis makinoi* | 13 | 510 |
| *Polyphlebium borbonicum* | 6 | 124 |
| *Polyphlebium endlicherianum* | 4 | 269 |
| *Ptisana salicina* | 11 | 165 |
| *Vaginularia paradoxa* | 14 | 63 |
| *Vandenboschia speciosa* | 281 | 286 |

| **order** | **species name** | **habit** | **morphology** | **gemmae** | **dataset** |
| --- | --- | --- | --- | --- | --- |
| Cyatheales | *Cibotium glaucum* | terrestrial | cordate | not clear | ecophys |
| Hymenophyllales | *Abrodictyum dentatum* | terrestrial | noncordate | yes | ftl |
| Hymenophyllales | *Callistopteris apiifolia* | terrestrial | strap-ribbon | yes | ftl |
| Hymenophyllales | *Callistopteris baldwinii* | terrestrial | strap-ribbon | yes | ftl |
| Hymenophyllales | *Callistopteris baueriana* | terrestrial | strap-ribbon | yes | ftl |
| Hymenophyllales | *Hymenophyllum pallidum* | epiphytic | noncordate | yes | ftl |
| Hymenophyllales | *Hymenophyllum wrightii* | epiphytic | strap-ribbon | yes | ftl |
| Hymenophyllales | *Polyphlebium borbonicum* | epiphytic | noncordate | yes | ftl |
| Hymenophyllales | *Polyphlebium endlicherianum* | epiphytic | noncordate | yes | ftl |
| Hymenophyllales | *Vandenboschia speciosa* | epiphytic | filamentous | yes | both |
| Marattiales | *Ptisana salicina* | terrestrial | cordate | no | ftl |
| Osmundales | *Todea barbara* | terrestrial | cordate | not clear | ecophys |
| Polypodiales | *Acrostichum aureum* | terrestrial | cordate | yes | ecophys |
| Polypodiales | *Adiantum latifolium* | terrestrial | cordate | no | ecophys |
| Polypodiales | *Adiantum trapeziforme* | terrestrial | cordate | no | ftl |
| Polypodiales | *Amauropelta balbisii* | terrestrial | cordate | no | ecophys |
| Polypodiales | *Antrophyum obovatum* | epiphytic | ribbon | yes | ftl |
| Polypodiales | *Antrophyum reticulatum* | epiphytic | noncordate | yes | ftl |
| Polypodiales | *Archigrammitis tahitensis* | epiphytic | noncordate | no | ftl |
| Polypodiales | *Arthropteris palisotii* | epiphytic | noncordate | no | ftl |
| Polypodiales | *Calymmodon orientalis* | epiphytic | noncordate | no | ftl |
| Polypodiales | *Campyloneurum brevifolium* | epiphytic | noncordate | not clear | ecophys |
| Polypodiales | *Cyclopeltis semicordata* | terrestrial | cordate | not clear | ecophys |
| Polypodiales | *Dennstaedtia bipinnata* | terrestrial | cordate | not clear | ecophys |
| Polypodiales | *Diplazium harpeodes* | terrestrial | cordate | no | ftl |
| Polypodiales | *Diplazium striatastrum* | terrestrial | cordate | not clear | ecophys |
| Polypodiales | *Goniopteris curta* | terrestrial | cordate | not clear | ecophys |
| Polypodiales | *Goniopteris nicaraguensis* | terrestrial | cordate | not clear | ecophys |
| Polypodiales | *Lomagramma tahitensis* | epiphytic | noncordate | no | ftl |
| Polypodiales | *Lomariopsis boninensis* | epiphytic | strap-ribbon | yes | ftl |
| Polypodiales | *Lomariopsis brackenridgei* | epiphytic | noncordate | no | ftl |
| Polypodiales | *Loxogramme parksii* | epiphytic | strap-ribbon | not clear | ftl |
| Polypodiales | *Microgramma reptans* | epiphytic | noncordate | not clear | ecophys |
| Polypodiales | *Nephrolepis biserrata* | epiphytic | cordate | no | ecophys |
| Polypodiales | *Phlebodium pseudoaureum* | epiphytic | cordate | not clear | ecophys |
| Polypodiales | *Pityrogramma calomelanos* | terrestrial | cordate | no | ecophys |
| Polypodiales | *Pleurosoriopsis makinoi* | epiphytic | strap-ribbon | yes | ftl |
| Polypodiales | *Pteris altissima* | terrestrial | cordate | not clear | ecophys |
| Polypodiales | *Thelypteris palustris* | terrestrial | cordate | not clear | ecophys |
| Polypodiales | *Vaginularia paradoxa* | epiphytic | not clear | not clear | ftl |

**Table S2**. A list of species in this study, their phylogenetic order, habit, morphology, presence of gemmae, and inclusion in the ecophysiology (ecophys) or niche expansion dataset (ftl), or both.
